# From repeating routes to planning novel routes: the impact of landmarks and ageing on route integration and cognitive mapping

**DOI:** 10.1007/s00426-020-01401-5

**Published:** 2020-09-14

**Authors:** Ramona Grzeschik, Christopher Hilton, Ruth C. Dalton, Irma Konovalova, Ella Cotterill, Anthea Innes, Jan M. Wiener

**Affiliations:** 1 Psychology Department, Ageing and Dementia Research Centre, Bournemouth University, Poole, UK; 2 Otto Von Guericke University, Magdeburg, Germany; 3 Lancaster School of Architecture, Lancaster University, Lancaster, UK; 4 Salford Institute for Dementia, University of Salford, Salford, UK

## Abstract

The integration of intersecting routes is an important process for the formation of cognitive maps and thus successful navigation. Here we present a novel task to study route integration and the effects that landmark information and cognitive ageing have on this process. We created two virtual environments, each comprising five places and one central intersection but with different landmark settings: in the Identical Landmark environment, the intersection contained visually monotonic features whereas the intersection contained visually distinctive features in the Different Landmarks environment. In both environments young and older participants were presented with two short routes that both traversed through the shared intersection. To test route integration, participants were asked to either repeat the learning routes, to navigate the routes from the destination to the starting place or to plan novel routes. As expected, results demonstrate better performance when repeating or retracing routes than when planning novel routes. Performance was better in younger than older participants and in the Different Landmark environment which does not require detailed knowledge of the spatial configuration of all places in the environment. A subgroup of the older participants who performed lower on a screening test for cognitive impairments could not successfully complete the experiment or did not reach the required performance criterion. These results demonstrate that strategically placed landmarks support the integration of route knowledge into spatial representations that allow for goal-dependent flexible navigation behaviour and that earliest signs of atypical cognitive ageing affect this process of route integration.

**Electronic supplementary material:**

The online version of this article (10.1007/s00426-020-01401-5) contains supplementary material, which is available to authorized users.

## Introduction

The integration of overlapping route knowledge has long been postulated to be a crucial process in the establishment of cognitive map-like spatial representations (Hanley & Levine, 1983; Montello, 1998; Siegel & White, 1975; Trullier, Wiener, Berthoz, & Meyer, 1997). In this study we present a novel task to investigate (1) this integration process and (2) the effects of cognitive ageing on the integration of route knowledge into cognitive maps.

Route knowledge is often conceptualised as a series of stimulus–response (S–R) associations in which the recognition of a place or landmarks triggers a movement response (e.g. “Turn left at gas station”; Waller & Lippa, 2007). Importantly, this type of knowledge allows navigators to only travel between the start location and one particular destination in the environment along a specific route. In other words, route knowledge does not allow for any goal-dependent flexibility such as choosing different actions at specific intersections depending on internal state or goal of the navigator.

Several studies investigating route learning suggest that route knowledge is more complex than simple S–R associations. Instead, route knowledge is best described as consisting of stimulus–response–stimulus (S–R–S) associations (Hilton et al., under review; Schinazi, & Epstein, 2010; Strickrodt, O’Malley, & Wiener, 2015). In contrast to ‘simple’ S–R associations, S–R–S associations allow for predicting or anticipating the next stimulus (i.e. place) from the current stimulus and the next action (see Trullier, Wiener, Berthoz, & Meyer, 1997).

**Fig. 1:**
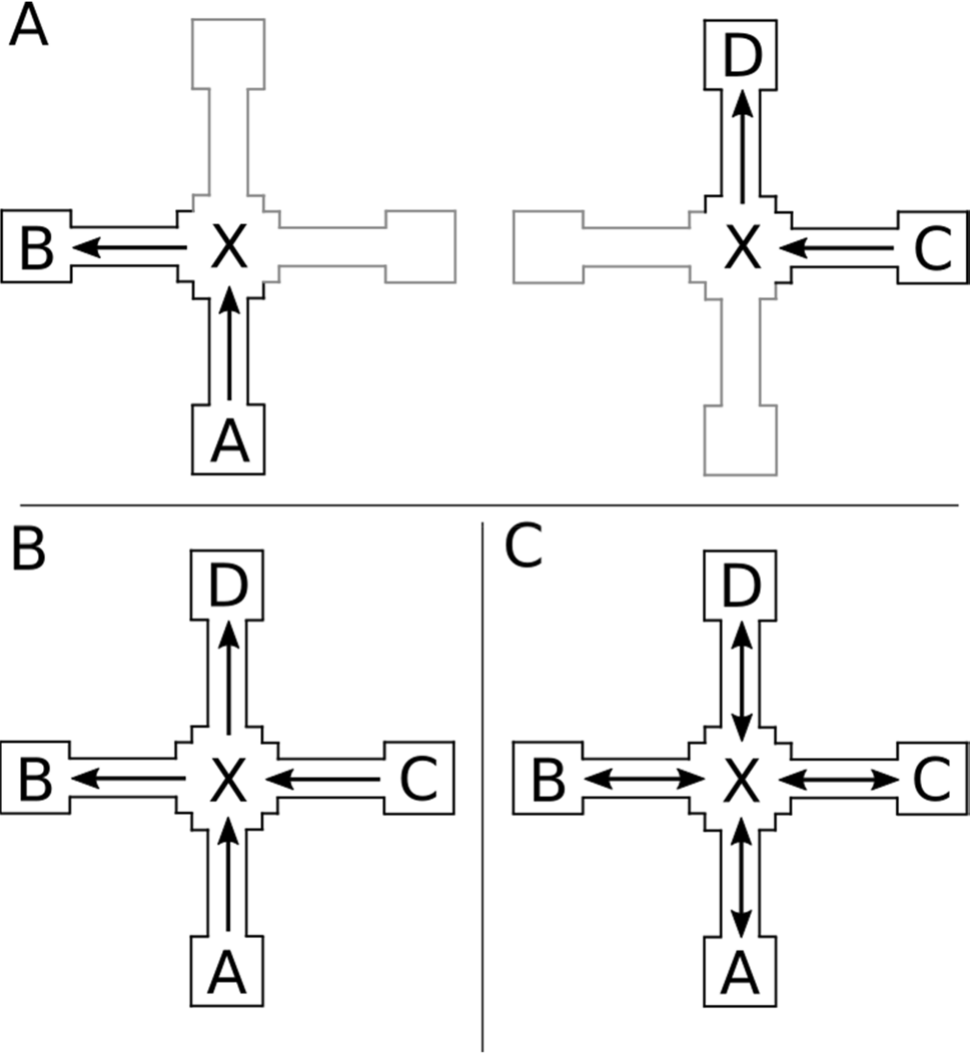
**a** Two short routes traverse through the same place (place X). **b** Integration of these two routes results in a spatial representation that describes the connectivity between all 4 places in the environment, thus allowing, for example, to navigate from the start of one route (e.g., place A) to the destination of the other route (e.g., place D); **c** if all places serve as start and goal places, the representation becomes entirely goal independent and can support navigation also from the destination of one route (e.g., place B) to the start of the other route (e.g., place C)

Being able to anticipate upcoming places on the basis of particular movement decisions is a prerequisite for goal-dependent flexible navigation behaviour as it allows the navigator to compare and choose between different actions and thus to plan different paths through the environment depending on the navigators’ goal (Trullier, Wiener, Berthoz, & Meyer, 1997). Flexible goal-dependent navigation behaviour can be achieved if independent routes, each leading from a unique start place to a unique goal place and traversing through a common place are merged into an integrated representation of space (see Fig. 1).

In its most basic form cognitive maps describe the topology of space, i.e. the connectivity between places and the actions required to move between neighbouring places (Franz & Wiener, 2008; Kuipers, 1978; Mallot & Gillner, 2000). Unlike configural map-like representations, purely topological representations of space are not metrically embedded (Chrastil & Warren, 2014). If every place in such topological representations can serve as a potential start place and destination (Fig. 1c), the representation supports true goal-dependent flexible navigation. That is, depending on the goal, navigators can choose different actions at places where the original routes intersect (hereafter intersections) and plan and navigate novel routes that have not been learned beforehand. In this study we take the ability to plan and navigate novel routes as an indication for the presence of a cognitive map-like integrated spatial representation.

Let us consider what the process of integrating route knowledge entails: Imagine two short routes that meet at a four-way intersection (see examples in Fig. 1): first, integration requires that navigators understand that different routes are traversing through the same place. Second, integration requires an understanding of the spatial relationship between the different paths leading from the four places to the intersection to create a correctly integrated representation. The difficulty of this second task depends on the information available at the intersection. If the intersection contains distinct visual features, such as landmarks, navigators can associate these with the different approach directions to facilitate their understanding of the spatial configuration (Wiener, de Condappa, Harris, & Wolbers, 2013, Wiener et al., 2019). Alternatively, the intersection may have a very similar visual appearance when approached from different directions. In this case, integration requires a good understanding of the spatial relationship of the relevant route segments. In the current study we address the question of how landmark information at the intersection affects route integration by asking participants to either navigate an environment in which the intersection contains distinct visual landmarks or an environment in which the intersection is visually identical when approached from different directions.

Age-related navigation deficits in navigation and spatial learning are well established (for a recent overview, see Lester, Moffat, Wiener, Barnes, & Wolbers, 2017). Cognitive ageing is associated with performance declines in both route learning tasks (Grzeschik, Conroy-Dalton, Innes, Shanker, & Wiener 2019; Head & Isom, 2010; Hilton, Miellet, Slattery, & Wiener, 2019) as well as in tasks that require cognitive mapping or cognitive map usage (Iaria, Palermo, Committeri, & Barton, 2009; Wolbers & Hegarty, 2010).

Route learning is typically associated with the use of egocentric reference frames, as the spatial information (e.g., “Turn left at gas station”) is dependent on the navigator’s position and orientation in space. Cognitive mapping or cognitive map usage, in contrast, is associated with allocentric reference frames and mechanisms, as they are independent of the navigator’s position in space (for an overview, see Wolbers & Wiener, 2014). Importantly, cognitive ageing is associated with larger impairments in tasks that cannot be solved by egocentric navigation strategies alone (for an overview, see Lester, Moffat, Wiener, Barnes, & Wolbers, 2017). A possible explanation for these findings is that the hippocampal circuit which is crucial for allocentric navigation mechanisms is particularly susceptible to age-related functional and structural changes (Raz et al., 2005).

Few earlier studies have specifically addressed how routes are integrated to form cognitive maps and how this process is affected by cognitive ageing. However, Wiener, de Condappa, Harris, and Wolbers, (2013), have investigated how cognitive ageing affects people’s ability to understand the spatial relationship between intersections and paths leading towards the intersections. As discussed above, this is a crucial component of cognitive mapping. Specifically, Wiener and colleagues asked young and older participants to learn a short route with four intersections. After each training phase, participants approached each intersection either from the same direction (same direction trials) as during training or from one of the other directions (different direction trials) and were asked to indicate the direction in which the route continued. Both age groups performed well on same direction trials which could be solved using egocentric spatial knowledge. The different direction trials, in contrast, required knowledge about the spatial relationship between the landmarks and the corridor in which the route continued and the older participants showed severely impaired performance in these trials. Results from this study suggest that cognitive ageing should affect route integration.

Given our limited understanding of the exact nature of the processes involved in integrating route knowledge into cognitive maps and the effect that cognitive ageing has on these processes the aims of the current study are as follows: First, to introduce a novel navigation task, designed to study the process of integrating route knowledge into cognitive map-like representations that allow for goal-dependent flexible navigation behaviour. Second, to study how landmark information available at intersections shared by multiple routes affects the integration of route knowledge. Finally, to study the effect of cognitive ageing on people’s ability to integrate route knowledge into cognitive maps.

In the experiment, participants were shown two short routes that both traversed through a shared intersection during encoding (similar to Fig. 1a). During the test trials, participants were then asked to either repeat the routes (route repetition trials), to navigate the routes from the destination to the starting place (retrace trials) or to navigate between all other combinations of starting place and destination of the original routes (novel route trials).

We expected best performance in repetition trials in which participants can rely on S–R(–S) associations formed during training. We expected route retracing trials to be harder than repetition trials as they require additional transformations or knowledge about the spatial relationship between the two paths and the intersection of the training routes (Allison & Head, 2017; Wiener, Kmecova, & de Condappa, 2012; Wiener et al., 2019). Finally, we expected novel route trials to be the most difficult trial types as these involve the planning of novel paths between origins and destinations which requires integrating knowledge from the training routes.

**Fig. 2:**
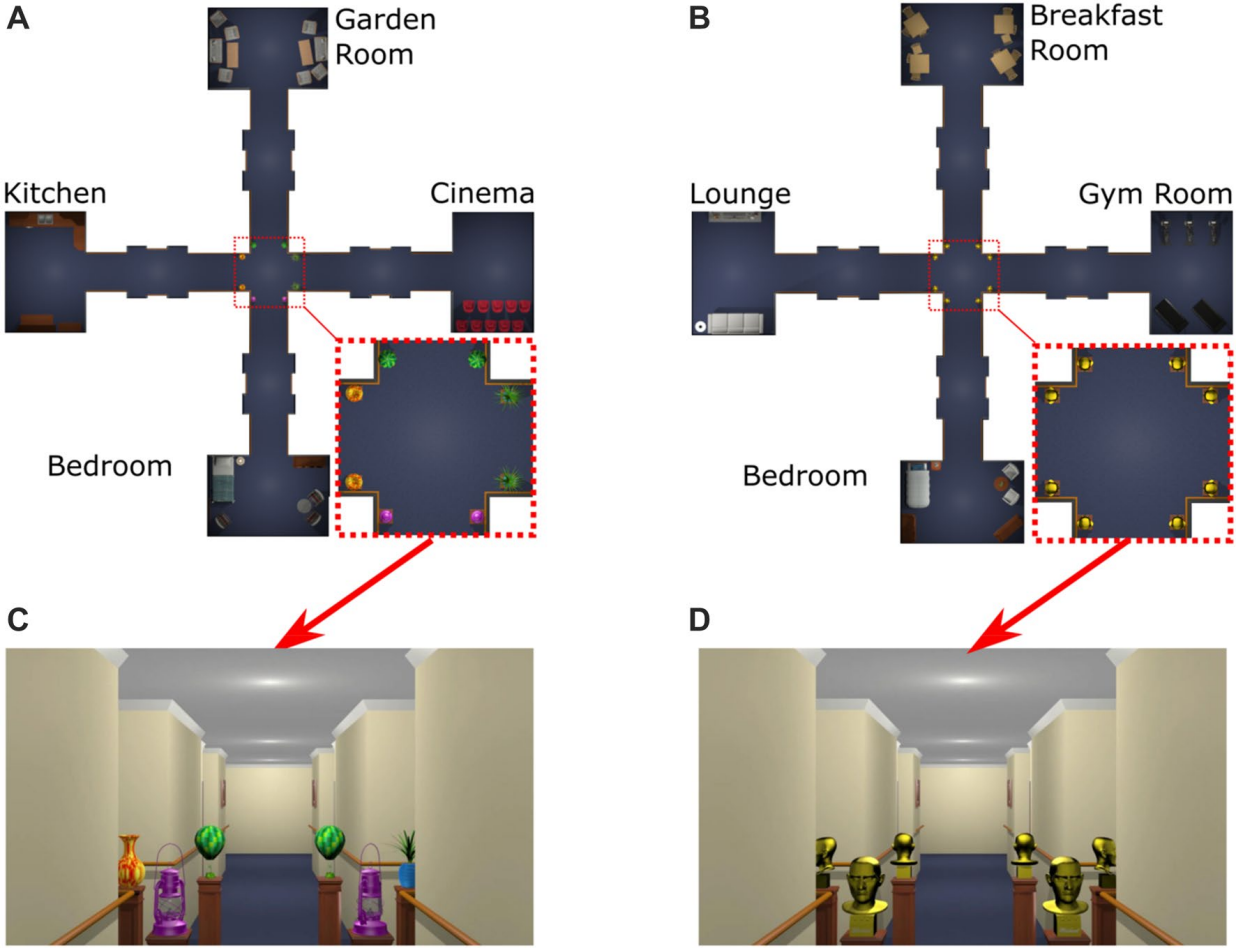
Layouts and screenshots of the environments. **a**, **c** *Different Landmarks* environment: four different objects were placed as sets of two at the entrances of each corridor in the central intersection; **b**, **d** for the *Identical Landmark* environment only one object was used

To address the question of how the layout of the intersection affects route integration, we designed two virtual environments. In the *Identical Landmark* environment, the intersection contained visually monotonic features such that it looked the same independent of the path from which it was approached (see Fig. 2d). In the *Different Landmarks* environment, the intersection contained visually distinctive features such that it looked different when approaching it from the different paths or directions (see Fig. 2c).

We expected route integration to be more difficult in the *Identical Landmark* environment than in the *Different Landmarks* environment. This should affect performance particularly in novel route trials which rely on integrated route knowledge. In the *Identical Landmark* environment, all the paths leading from the intersection to the rooms look identical. To integrate the two different routes correctly, participants therefore had to understand the exact spatial relationship between all five places in the environment. In the *Different Landmarks* environment, landmark information enables participants to visually distinguish the four paths leading from the intersection to the remaining four rooms. This landmark information should facilitate the integration of the two routes and could support planning of novel routes even without an exact understanding of the spatial relationship between all five places in the environment.

During route integration, directional S–R–S associations (Schinazi & Epstein, 2010; Strickrodt, O’Malley, & Wiener, 2015) need to be transformed into a topological representation that supports flexible navigation in any direction (see Fig. 1b, c). If this was a gradual process, we expected better performance for novel routes in which the destination was also a destination in the training route and worse performance for novel routes in which the destination was a start place in the training route.

Finally, and in line with earlier research (see Lester, Moffat, Wiener, Barnes, & Wolbers, 2017), we expected age-related declines in navigation performance, particularly in navigating retrace and novel route trials which cannot be solved using egocentric route navigation strategies. Importantly, we expected these age-related performance differences to be more pronounced in the *Identical Landmark* environment which requires an exact understanding of the spatial relationship between all five places.

## Material and methods

### Virtual environment

#### Apparatus

The experiment was programmed using Unity (Unity Technologies, San Francisco, USA) and presented on a portable Tablet PC (Huawei Mediapad M2) with a 10″ display.

#### Virtual environment

We created two environments, the *Different Landmarks* and the *Identical Landmark* environment, using 3D Studio Max (Autodesk Inc., San Rafael, USA). Both environments had the same basic layout consisting of five places, four rooms and corridors connected by a cross-shaped intersection. In the *Different Landmark* environment different objects were placed at the central intersection at each corridor entrance (Fig. 2a, c). In the *Identical Landmark* environment, the same object was placed at the central intersection at the entrance of each corridor (Fig. 2b, d). The environments were created to resemble a residential development or care home and were designed to look as naturalistic as possible. Nevertheless, the pictures on the walls in the different corridors were all identical which ensured that they could not be used as landmarks to support navigation.

### Participants

A total of 119 participants with normal or corrected-to-normal vision (52 younger adults [30 females; mean age 22.12 ± 3.75 years; range 18–33] and 67 older adults [39 females; mean age 70.87 ± 4.57 years; range 65–85]) took part in the experiment. Overall cognitive functions were assessed with the MoCA (Montreal Cognitive Assessment; Nasreddine et al., 2005). Most of the younger participants were Psychology undergraduates at Bournemouth University and were rewarded course credits for their participation. The older participants were volunteers and received monetary compensation for their participation in the study. Ethical approval was obtained from the Science, Technology and Health Research Ethics Panel at Bournemouth University and written informed consent was obtained from all participants, in accordance with the Declaration of Helsinki (World Medical Association, 2000).

### Procedure

Participants were tested one at a time in a quiet room in the Psychology Department at Bournemouth University. After they signed the consent form, the experimenter administered the MoCA. Participants were then handed a tablet PC that they were free to either hold in their hands or to place on a desk in front of them and were informed about the nature of the experiment. Participants were explicitly instructed that they would be shown short routes during the learning phase and that they had to recall these routes or plan novel routes during the test phase (see details below).

The experiment comprised a maximum of eight experimental sessions or until participants reached over 90% performance in a single session. This criterion was chosen to ensure that participants had very good knowledge of the environment before the experiment was ended, while allowing for a single error without prolonging the experiment unnecessarily. Each experimental session consisted of two training phases and two test phases (see Table 1 for details).

**Table 1:** Exemplary experimental session showing the sequence of training and test trials for both environments

Different Landmarks				Identical Landmark			
Start	Destination	Training/test	Route type	Start	Destination	Training/test	Route type
Bed room	Kitchen	Training		Lounge	Bed room	Training	
Cinema	Garden R	Training		Breakfast R	Gym room	Training	
Garden R	Kitchen	Test	Novel	Lounge	Gym room	Test	Novel
Cinema	Garden R	Test	Repeat	Gym room	Breakfast R	Test	Retrace
Bed room	Cinema	Test	Novel	Breakfast R	Bed room	Test	Novel
Cinema	Kitchen	Test	Novel	Bed room	Breakfast R	Test	Novel
Kitchen	Bed room	Test	Retrace	Gym room	Bed room	Test	Novel
Kitchen	Cinema	Test	Novel	Breakfast R	Gym room	Test	Repeat
Bed room	Kitchen	Training		Lounge	Bed room	Training	
Cinema	Garden R	Training		Breakfast R	Gym room	Training	
Garden R	Cinema	Test	Retrace	Breakfast R	Lounge	Test	Novel
Kitchen	Garden R	Test	Novel	Bed room	Gym room	Test	Novel
Garden R	Bed room	Test	Novel	Lounge	Bed room	Test	Repeat
Bed room	Garden R	Test	Novel	Gym room	Lounge	Test	Novel
Cinema	Bed room	Test	Novel	Lounge	Breakfast R	Test	Novel
Bed room	Kitchen	Test	Repeat	Bed room	Lounge	Test	Retrace

Each session consisted of two training and two test phases

*Training Phase 1* comprised passive transportation at walking speed (1.1 m/s with a camera height of 1.65 m) along two training routes. The training routes always contained a left or right turn at the intersection and each of the four places surrounding the common intersection was either a start place or a destination for one of the routes.

**Fig. 3:**
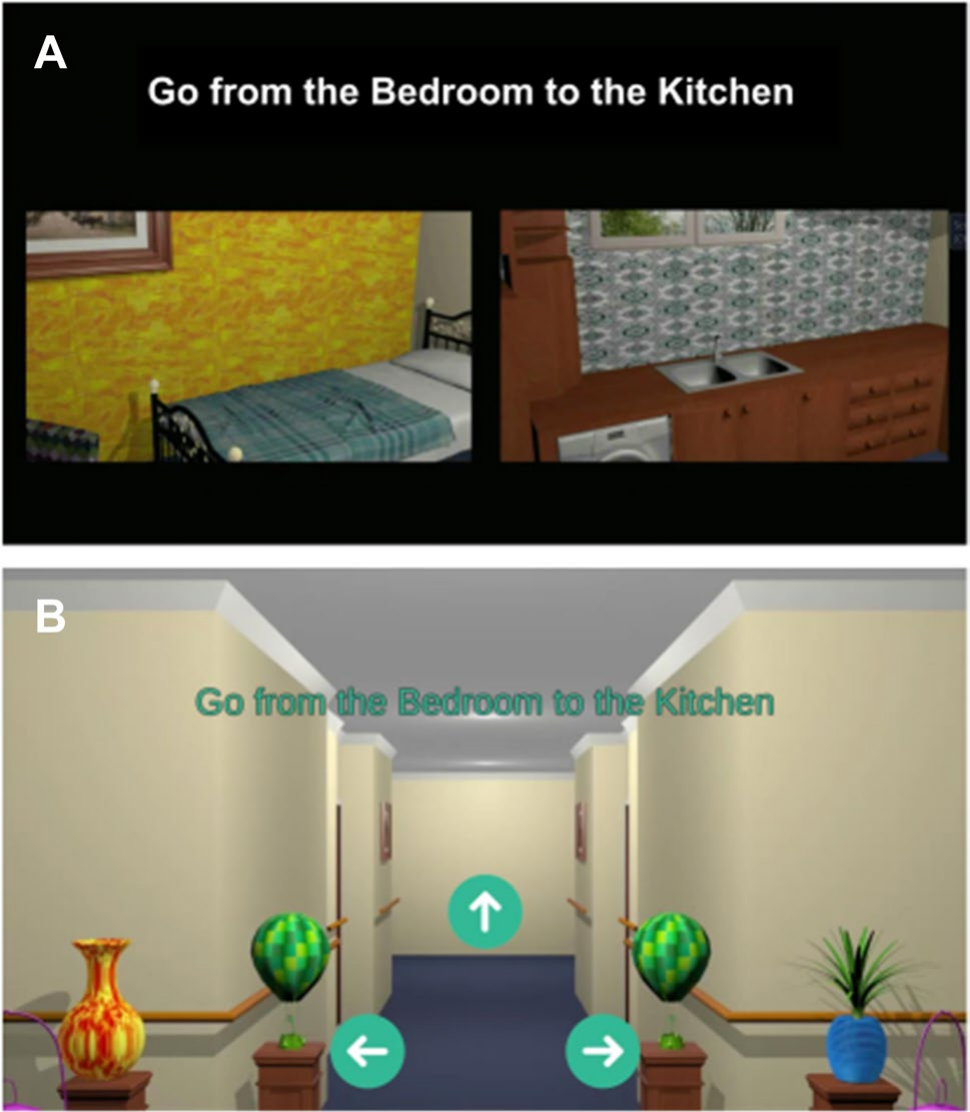
Exemplary task in the *Different Landmarks* condition. **a** Participants were first shown the route that they have to go. **b** When they approached the intersection, the video would stop and the task would be shown for a second time. Now they are asked to indicate the correct direction by pressing one of the arrows

*Test Phase 1* comprised a random selection of six of the 12 test trials (2 repetition trials [identical to the training routes], 2 retracing trials [training routes in opposite direction] and 8 novel routes [routes that use the 4 rooms as either start or destination in all the remaining possible combinations], see Table 1 for details). At the beginning of a test trial participants were presented with instructions for the specific navigation task (e.g. “Go from the bedroom to the kitchen” see Fig. 3a) and then passively transported from the start place to the intersection where they were asked to indicate the direction to the goal (Fig. 3b). Participants reported their decisions by tapping on the corresponding arrow on the tablet, but they did not receive feedback to avoid learning during the test session.

*Training Phase 2* was identical to training phase 1.

*Test Phase 2* was identical to test phase 1, but used the remaining 6 test trials not used in test phase 1.

Participants’ performance was calculated at the end of each experimental session. The experiment continued until participants responded correctly for at least 11 of the 12 test trials or until the 8th experimental session was completed.

About one half of the participants (25 younger and 34 older adults) were tested in the *Different Landmarks* environment (*Different Landmarks condition*) and the other half of the participants were tested in the *Identical Landmark* environment (*Identical Landmark condition*).

## Results

All of the 52 younger participants completed the experiment, whilst 20 of the 67 older participants did not finish the experiment (i.e. did not reach performance criterion and did not complete eight experimental sessions). We did not formally collect data on the reasons why participants decided to withdraw, however informal discussions suggested that they found the task too difficult. More older participants withdrew from the *Identical Landmark* condition (12/20) compared to the *Different Landmarks* condition (8/20), however a chi-squared test revealed this distribution did not significantly differ from chance (*p* = 0.371).

Of the participants who completed the experiment, one of the 52 younger participants and ten of the 47 older participants did not reach the criterion. A chi-squared test showed that this distribution of older and younger adults not reaching criterion was significantly different from chance (*p* = 0.007). In total, there were 51 younger and 37 older participants who reached criterion. Participants’ demographic data (Age, MoCA, Gender) for each group is summarised in the Supplementary Material (Tables Suppl. 1–Suppl. 4).

### Number of sessions to reach criterion

**Fig. 4:**
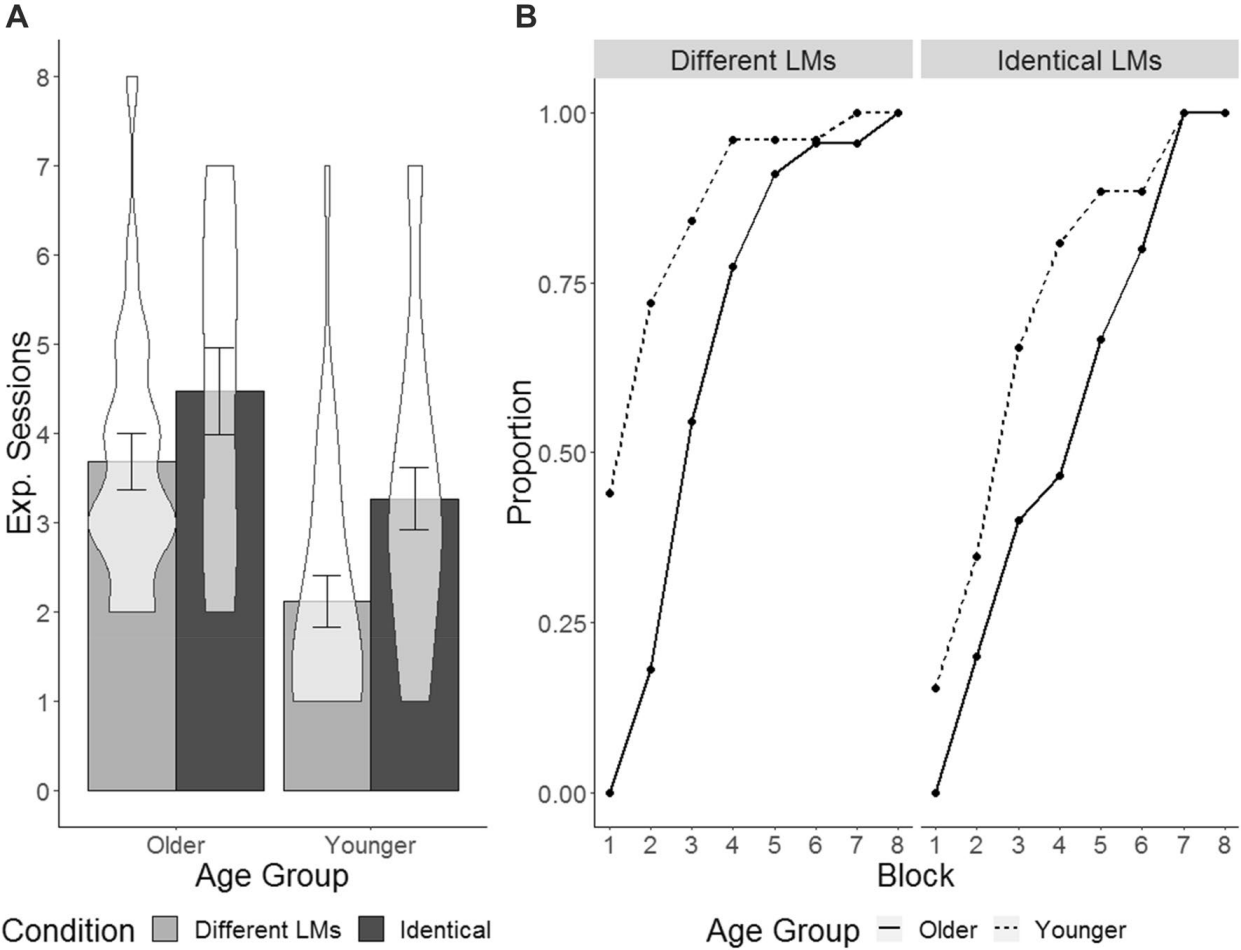
Average number of sessions to reach criterion (**a**) and cumulative proportion of participants to reach criterion in each block (**b**). The error bars represent standard error of the mean, the shaded areas in the bar plots show the probability density of the data at different values

For the participants who reached criterion, we conducted a between-groups ANOVA with the factors age group (younger, older) and condition (*Identical Landmark*, *Different Landmarks*). There were significant main effects of age (*F*(1,84) = 15.54, *p* < 0.001, $\eta_{{\text{p}}}^{{2}}$ = 0.016) and condition (*F*(1,84) = 8.10, *p* = 0.005, $\eta_{{\text{p}}}^{{2}}$ = 0.009), but no significant interaction (*F*(1,84) = 0.26, *p* = 0.611, $\eta_{{\text{p}}}^{{2}}$ = 0.003). Specifically, older adults took more sessions to reach criterion than younger adults (4.00 vs 2.71) and participants took more sessions to reach criterion in the *Identical* compared to the *Different Landmarks* condition (3.71 vs 2.85; see Fig. 4).

### Performance for different route types

When comparing performance between route types, we did not include session as a factor because participants completed a variable number of sessions depending on how long it took them to reach criterion. We analysed data from the younger and older adults who reached criterion, as well as the older participants who did not reach criterion (either because they did not finish the experiment or because they reached the maximum number of attempts). Since the older participants who did not reach criterion are qualitatively different from the older adults who did, they were included as a separate group which allowed us to investigate possible reasons for why they did not reach criterion. The participant groups in the following analysis were: Y+ (younger participants who reached criterion), O+ (older participants who reached criterion), and O− (older participants who did not reach criterion). We did not include Y− since there was only one younger participant who did not reach criterion. Tables with average performance for route type by condition and by participant group, and the number of trials attempted are included in the Supplementary Material (tables Suppl. 5, Suppl. 6).

An ANOVA with the between factors group (Y+, O+, O−), and condition (*Identical Landmark, Different Landmarks*) and the within factor route type (repeat, retrace, novel route) revealed significant main effects of group (*F*(2,112) = 66.04, *p* < 0.001, $\eta_{{\text{p}}}^{{2}}$ = 0.541) and route type (*F*(2,224) = 42.89, *p* < 0.001, $\eta_{{\text{p}}}^{{2}}$ = 0.277) on performance, while there was no main effect of condition (*F*(1,112) = 0.96, *p* = 0.331, $\eta_{{\text{p}}}^{{2}}$ = 0.008).

Specifically, performance significantly differed between all three groups (Y+ vs. O+ [76.31 vs. 63.23%, *t*(85.09) = 4.14, *p* < 0.001, *d* = 0.84]; O+ vs. O− [63.23 vs. 36.68%; *t*(64.89) = 10.50, *p* < 0.001, *d* = 2.51]; Y+ vs O− [76.31 vs. 36.68%; *t*(77.56) = 13.19, *p* < 0.001, *d* = 2.59]). Performance for repeat and retrace routes was better than performance for novel routes (repeat vs novel route [76.15 vs. 56.71%; *t*(231.92) = 6.48, *p* < 0.001, *d* = 0.84]; retrace vs novel route [69.81 vs 56.71%; *t*(227.72) = 3.81, *p* < 0.001, *d* = 0.50], while performance difference between repeat and retrace did not reach statistical significance (76.15 vs. 69.81%; *t*(219.53) = 1.92, *p* = 0.057, *d* = 0.25).

There were significant interactions between route type and condition (*F*(2,224) = 5.47, *p* = 0.005, $\eta_{{\text{p}}}^{{2}}$ = 0.047), and route type and group (*F*(4,224) = 4.83, *p* < 0.001, $\eta_{{\text{p}}}^{{2}}$ = 0.079). The interactions between group and condition (*F*(2,112) = 1.84, *p* = 0.16, $\eta_{{\text{p}}}^{{2}}$ = 0.032) and the three-way interaction between group, condition and route type (*F*(4,224) = 0.31, *p* = 0.87, $\eta_{{\text{p}}}^{{2}}$ = 0.006) were not significant.

**Fig. 5:**
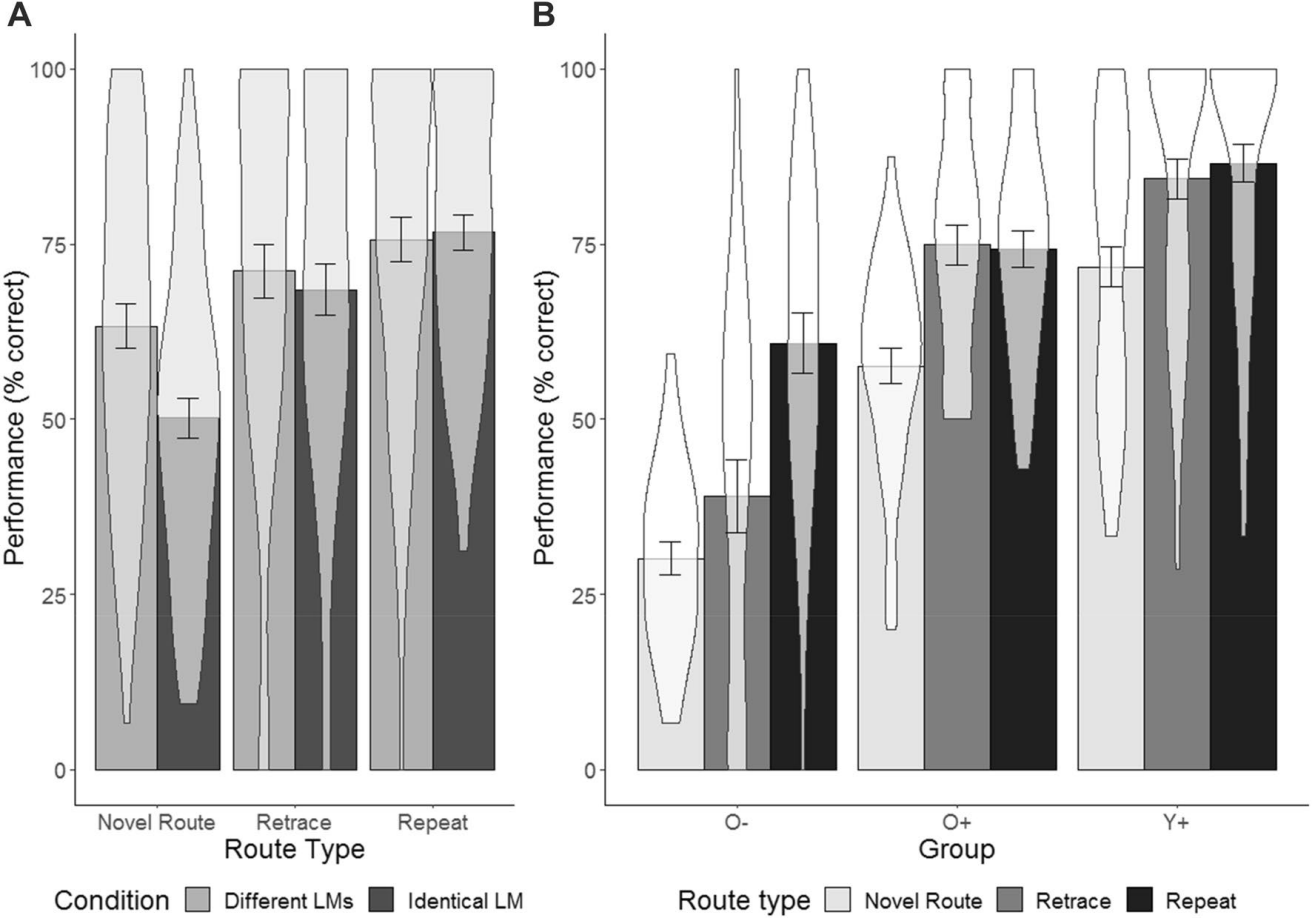
Performance for each route type between conditions (**a**); performance for each route type for the different age groups (**b**). The error bars represent standard error of the mean, the shaded areas in the bar plots show the probability density of the data at different values

To follow up the interaction between route type and condition we compared performance between conditions for each route type using independent *t* tests. Significance was assessed using the Bonferroni-corrected alpha level of 0.017. There was no significant difference between conditions for the repeat routes (*t*(111.28) = 0.25, *p* = 0.80, *d* = 0.05) or the retrace routes (*t*(115.76) = 0.50, *p* = 0.618, *d* = 0.09). For the novel routes, participants in the *Different Landmarks* condition performed significantly better than participants in the *Identical Landmark* condition (63.27 vs 50.17%; *t*(114.49) = 3.06, *p* = 0.003, *d* = 0.56; see Fig. 5).

To follow up the interaction between route type and group we conducted independent *t* tests between each route type separately for each group. Significance was assessed using the Bonferroni-corrected alpha level of 0.006. Results of the *t* tests are presented in Table 2 and show that the O− group performed significantly worse in retrace routes compared to the repeat route (38.98 vs 60.85%), whereas there was no significant difference between these route types for the O+ and Y+ groups.

**Table 2:** Follow up *t* tests for the route type × group interaction

Group	Repeat vs novel			Repeat vs retrace			Retrace vs novel		
	*t*	*p*	*d*	*t*	*p*	*d*	*t*	*p*	*d*
Y+	*t*(99.29) = 3.73	< 0.001	0.74*	*t*(99.54) = 0.57	0.569	0.11	*t*(99.93) = 3.06	0.003	0.61*
O+	*t*(71.97) = 4.62	< 0.001	1.07*	*t*(71.52) = − 0.16	0.873	0.03	*t*(71.27) = 4.58	< 0.001	1.06*
O−	*t*(44.80) = 6.24	< 0.001	1.61*	*t*(56.24) = 3.24	0.002	0.84*	*t*(40.53) = 1.56	0.127	0.40

Note that the degrees of freedom are corrected for inhomogeneity of variances

Note that the difference in performance between the novel routes and the repeat or retrace routes were numerically larger in the O+ than the Y+ group (see Fig. 5b and *t* values for these comparisons in Table 2). To investigate whether this effect contributed to the route type × group interaction we reran the ANOVA without the O− group. This analysis, however, did not render a significant route type × group interaction (*F*(2,168) = 1.02, *p* = 0.36, $\eta_{{\text{p}}}^{{2}}$ = 0.012).

### Origins and destinations

To analyse whether novel routes with destinations that were also destinations in the training phase rendered better performance than novel routes with destinations that were start places in the training phase, we divided the novel routes into two destination groups: congruent (destination in test phase was also a destination in training phase) and incongruent (destination in test phase was an origin in training phase).

An ANOVA with the within factor destination group (congruent, incongruent) and the between factors group (Y+, O+, O−) and condition (identical, different) replicated the significant main effects of group (*F*(2,112) = 57.88, *p* < 0.001, $\eta_{{\text{p}}}^{{2}}$ = 0.508) and condition (*F*(1,112) = 12.16, *p* < 0.001, $\eta_{{\text{p}}}^{{2}}$ = 0.098) that we described above. Importantly though, there was no main effect of destination group (*F*(1,112) = 1.51, *p* = 0.222, $\eta_{{\text{p}}}^{{2}}$ = 0.013) and the difference in performance between trials with congruent and incongruent destinations was less than 2% (congruent: 55.82%; incongruent: 57.50%). None of the interactions were significant (condition × group: *F*(2,112) = 2.33, *p* = 0.10, $\eta_{{\text{p}}}^{{2}}$ = 0.040; condition × destination group: *F*(1,112) = 2.38, *p* = 0.13, $\eta_{{\text{p}}}^{{2}}$ = 0.021; group × destination group: *F*(2,112) = 0.54, *p* = 0.59, $\eta_{{\text{p}}}^{{2}}$ = 0.009; condition × group × destination group: *F*(2,112) = 1.14, *p* = 0.32, $\eta_{{\text{p}}}^{{2}}$ = 0.020).

### MoCA

**Fig. 6:**
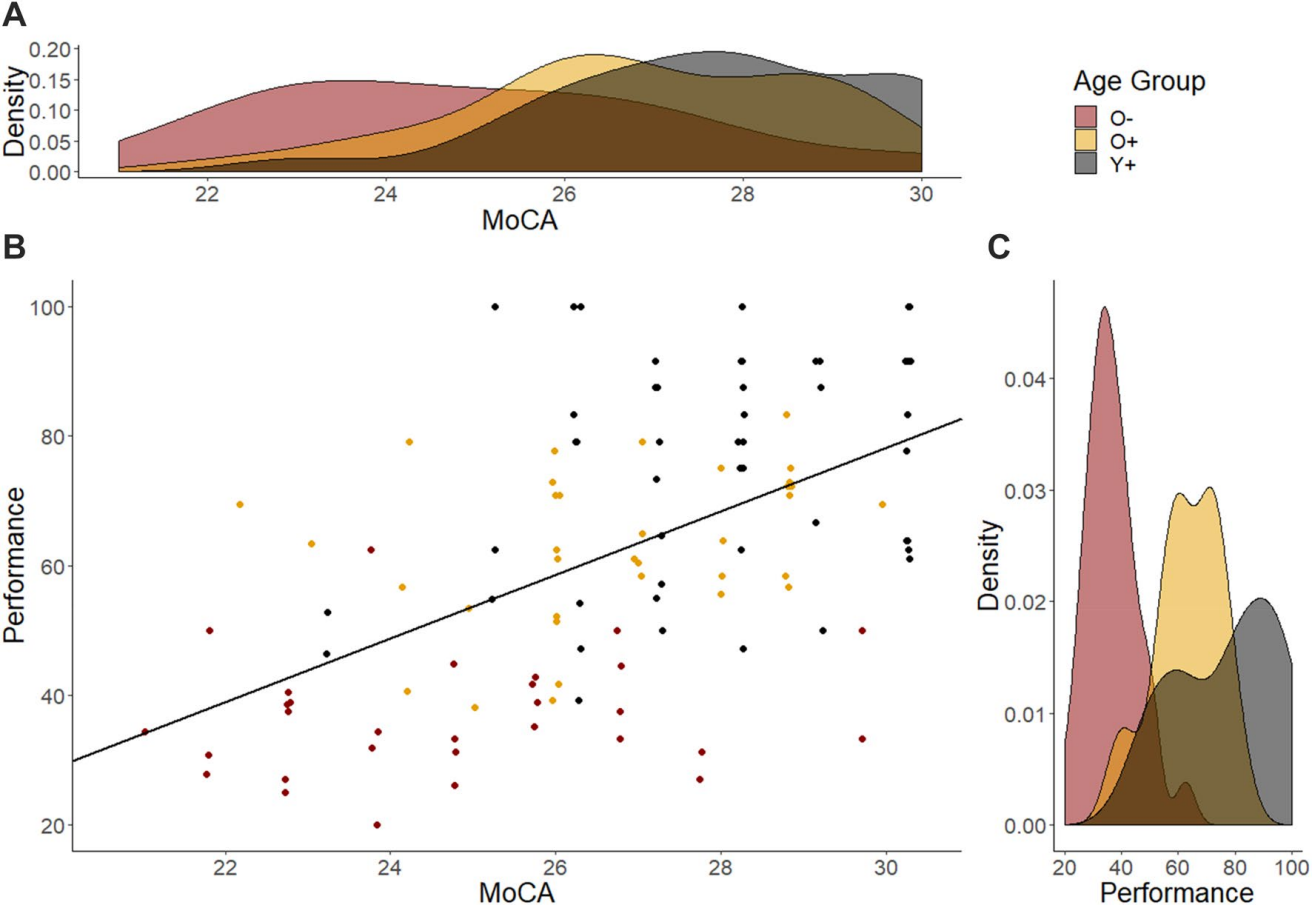
Relationship between performance and MoCA scores with regression slope (**b**), and distribution of MoCA scores (**a**) and performance (**c**) for each of the three participant groups

We analysed participant MoCA scores as a post-hoc investigation into why the O− group experienced greater difficulty with the task compared to the O+ and Y + groups. A between-groups ANOVA with the factor of group revealed a significant main effect of group (*F*(2,115) = 17.79, *p* < 0.001, $\eta_{{\text{p}}}^{{2}}$ = 0.236; see Fig. 6a). Follow up independent *t* tests revealed that the O− group had significantly lower scores than the O+ group (24.97 ± 2.34 vs. 26.81 ± 1.93; *t*(56) = −3.47, *p* = 0.001, *d* = 0.87) and the Y+ group (24.97 ± 2.34 vs. 27.71 ± 1.83; *t*(49.76) = −5.50, *p* < 0.001, *d* = 1.35). The Y+ group had higher MoCA scores than the O+ group (*t*(75.19) = 2.20, *p* = 0.031, *d* = 0.48), however this effect did not survive Bonferroni correction of the alpha level to 0.017.

To investigate the relationship between MoCA score and performance, we conducted a multiple linear regression with the outcome variable of performance and the predictor variables of age and MoCA score. A significant regression equation was found (*R*^2^ = 0.42, *F*(3,114) = 30.40, *p* < 0.001) which revealed that MoCA score was a significant predictor of performance (*β* = 4.29, *t* = 2.41, *p* = 0.018; see Fig. 6b). There was no significant effect of age (*β* = 0.04, *t* = 0.05, *p* = 0.957) and no significant interaction between age and MoCA (*β* = − 0.02, *t* = 0.53, *p* = 0.596).

## Discussion

We used a novel task to study the integration of route knowledge into cognitive map-like spatial knowledge and the effects that cognitive ageing has on this process. In the experiment, participants were shown two separate routes that traversed through the same intersection and were then asked to (1) repeat these routes, (2) to retrace the routes from the goal to the start, and (3) to plan novel routes. This procedure was repeated until participants could successfully navigate the environment, i.e. until they reached an average performance of greater than 90% in one experimental session, or until they had completed eight experimental sessions. To study how landmark information at the central intersection influenced the integration of route knowledge, we designed two environments. In the *Identical Landmark* environment, the intersection had the same visual appearance independent from the path (direction) it was approached from. In the *Different Landmarks* environment, in contrast, different landmarks were associated with the different approach directions.

In line with our expectations, participants reached the performance criterion earlier in the *Different* than the *Identical Landmark* environment, demonstrating that the landmarks at the central intersection supported learning of the environment and thus successful navigation. While it is well known that landmarks can support navigation (Jansen-Osmann, 2002; Ruddle, Volkova, Mohler, & Bülthoff, 2011; Waller & Lippa, 2007), the analysis of performance by route type allows for insights into how landmarks support navigation in our task.

Planning and navigating a novel route, i.e. a route that has not been learned, requires participants to integrate and combine spatial knowledge acquired when learning different routes. It is exactly these types of navigation tasks (planning novel routes) that participants in the *Identical Landmark* environment struggled with more than participants in the *Different Landmarks* environment. Performance on the other types of navigation tasks (repeating or retracing routes), which can be solved by simpler mechanisms that do not require an integrated representation, was similar between environments. This result pattern suggests that the landmarks at the central intersection specifically supported the integration of routes.

The landmarks in the *Different Landmarks* environment were arranged such that each of the corridors leading to a room featured a unique landmark. If participants learned these associations between landmark and room, they could have used this knowledge to plan and successfully navigate novel routes. For example, if participants associated the yellow vase at the intersection with the kitchen (see Fig. 2a, c) they could simply move towards the yellow vase whenever the target destination of the navigation task was the kitchen. In this example, the landmark essentially functions as a beacon, as movement towards the landmark brings the navigator closer to the destination (Waller & Lippa, 2007). Importantly, the associations between landmarks at the central intersection and the rooms provide the topological knowledge that makes it possible to navigate between any room in the environment without the need for an exact understanding of the spatial configuration of the rooms. This is not possible in the *Identical Landmark* environment in which all corridors looked identical from the central intersection. In this case, participants could not plan novel routes, which always connected two places experienced on different learning routes, without understanding the configuration of the four rooms and the central intersection. Participants needed more sessions to reach criterion in the *Identical Landmark* environment in which configural knowledge is required than in the *Different Landmark* environment in which simple associations between landmarks and locations can support navigation. We believe that this difference in learning performance supports the idea that participants developed different spatial representations in the two environments.

It is important to stress at this point that participants who reached the criterion did successfully integrate route knowledge in both conditions. Even if the structure and content of the spatial representation differed, as suggested above (associations/topology vs. configurational knowledge), participants achieved goal-dependent flexible navigation behaviour at the central intersection in both conditions. Future research will have to further investigate and compare the exact nature of the spatial representations that participants developed in the *Identical Landmark* environment and the *Different Landmark* environment.

In contrast with earlier research (Allison & Head, 2017; Wiener, Kmecova, & de Condappa, 2012) and our expectations, we found that performance when repeating and retracing routes was comparable. In earlier studies we have argued that route retracing is not achieved by simply mirroring movement decisions at single decision points, but instead requires knowledge about the spatial relationship between neighbouring places along the route which makes it a more difficult task than repeating a route (Allison & Head 2017; Wiener, Kmecova, & de Condappa, 2012, Wiener et al., 2019). A likely reason for the lack of differences between route repetition and route retracing in the current study is that the routes were so short. Our routes were the shortest and simplest possible routes, requiring only a single decision. It is possible that this makes the strategy of simply mirroring a movement decision when retracing more prevalent. Alternatively, it may make memorising and using the spatial relationship between the corridors during retracing easier, as participants only have to remember two movement decisions (one for each training route).

Interestingly, performance when navigating novel routes did not depend on whether the destination served as an origin or a destination place in the training routes. This complements the finding that route repetition and route retracing rendered similar performance, thus suggesting that all places in the environment can serve as start locations as well as destinations, a prerequisite for spatial representations that support flexible navigation behaviour (Trullier, Wiener, Berthoz, & Meyer, 1997).

Our older participants needed longer to reach the performance criterion than our younger participants. In other words, they needed longer to learn the environment such that they achieved > 90% performance on all route types. This result was predicted and it is in line with earlier research that showed age-related deficits in forming spatial representations and navigation deficits (for an overview, see Lester, Moffat, Wiener, Barnes, & Wolbers,, 2017).

As discussed above, successful navigation in the *Identical Landmark environment* requires knowledge of the exact spatial configuration of the places in the environment. As earlier research suggests that cognitive ageing affects the formation of such configural spatial knowledge (Iaria, Palermo, Committeri, & Barton, 2009; Wiener, de Condappa, Harris, & Wolbers, 2013; for an overview, see Colombo et al., 2017). We therefore predicted that our older participants would have particular difficulties with learning the *Identical Landmark* environment which should have resulted in an interaction between age group and type of environment. In contrast to our predictions, however, we did not observe this interaction. Instead, we found similar age-related differences in the number of sessions required to reach the performance criterion in both environments. A possible explanation for the lack of the predicted interaction comes from the specific design and performance measures used in this study.

The requirement to reach > 90% performance within eight experimental sessions essentially selected for participants who were able to efficiently learn the environment. Almost 45% of our older participants either did not reach criterion (10/67) or did not finish the experiment (20/67) and were therefore not included in the ‘performance criterion’ analysis. In contrast, only one of our younger participants (2%) did not reach criterion. While we have not systematically recorded reasons for withdrawing from the experiment, some of our older participants who did not finish the experiment said that they found the task too difficult. Excluding older adults who did not reach criterion from the ‘performance criterion’ analysis may explain why we did not observe the predicted interaction between age group and environment type. It is important to note, however, that we observed a highly significant age difference, despite essentially selecting for good navigators.

To further investigate the impact of cognitive ageing on route integration and to develop a better understanding of how older adults who did not reach performance criterion differed from the rest, we included all participants in an analysis of performance across the different route types. Specifically, we split our participants into three groups: younger participants who reached criterion (Y+), older participants who reached criterion (O+) and those older participants who did not reach criterion (O−). The Y+ group outperformed the O+ group on all route types, which is reflecting the results of the criterion analysis. In line with the interpretation that older adults who did not finish the experiment found the experiment too hard, the O− group performed substantially weaker than the O+ or Y+ group. Interestingly, the O− group was the only group that showed performance differences between the repetition and retracing trials. Performance for route retracing and novel route trials was at chance level for the O− group. These results demonstrate that the O− group, i.e. the group of older participants who have not reached criterion or did not finish the experiment, had particular problems with those navigation trials that either require additional mental transformation (retracing trials) or an integrated representation of space (novel trials).

How can we explain the performance differences between the two older age groups who reached (O+) and did not reach performance criterion (O−)? We used the MoCA (Montreal Cognitive Assessment), an established screening tool for Mild Cognitive Impairment (MCI) to assess participants’ cognitive functioning. A comparison of MoCA scores between participant groups showed that our O− group had significantly lower MoCA scores than the other two participant groups. This demonstrates that the MoCA is sensitive for early signs of cognitive decline that affect navigation performance (c.f., O’Malley, Innes, & Wiener, 2018). This interpretation is further supported by the finding that MoCA scores were a significant predictor of overall navigation performance across all participant groups. Importantly, the model showed that age (which did not differ between the O− and O+ groups) did not explain the variance in performance when MoCA scores were included as a predictor. These results support the differential impact of ageing with typical or atypical trajectories on navigation ability (c.f. Cushman, Stein, & Duffy, 2008; O’Malley, Innes, & Wiener, 2018). Indeed, navigation tasks have more recently been suggested to be particularly sensitive markers of MCI- and AD-related cognitive decline (Cushman, Stein, & Duffy, 2008; Tu et al., 2015; Zygouris et al., 2017; for reviews see Cogné et al., 2017 and Coughlan, Laczó, Hort, Minihane, & Hornberger, 2018).

The results from the current study also have implications for the MoCA cut-off used to differentiate healthy ageing from MCI. Some studies have suggested cut-offs as low as 21/30 or 22/30 (Freitas, Simões, Alves, & Santana, 2013; Lee et al., 2008), while other studies suggest a higher cut-off of 26/30 (Nasreddine et al., 2005). Our results suggest that lower cut-offs may be too lenient and studies using such low cut-offs (e.g. Wiener, de Condappa, Harris, & Wolbers, 2013) may be overestimating the effects of typical or healthy ageing on navigation (c.f. O’Malley, Innes, & Wiener, 2018).

## Summary

We have introduced a novel navigation task to study how intersecting routes are integrated and how landmarks at the common intersection support the integration process. Our results show that landmarks do facilitate the integration of routes. We believe that landmarks at the central intersection are associated with the different rooms. They thus provide the topological knowledge that makes it possible to plan and navigate novel routes without the need for knowledge of the exact spatial configuration of the places within the environment.

While our young participants performed better than our older participants, both age groups benefited from the presence of distinctive landmarks. Importantly, we found that a subgroup of our older participants could not finish the experiment or did not reach the required performance criterion. This subgroup had lower MoCA scores than the remaining older participants, which suggests that the task we introduced here is sensitive to the earliest signs of cognitive impairment.

Finally, it is important to mention that our approach to study route integration and cognitive mapping differs from other approaches in which participants first learn two independent routes and are then informed about a connecting route (Ishikawa & Montello, 2006; Schinazi et al., 2013). In addition, instead of assessing participants’ knowledge of the spatial relationship between landmarks encountered on different routes by distance or direction judgments, we assessed their ability to navigate between places experienced on different routes. In other words, we focus on learning the topological relationships between places rather than their metric relationships. We believe that topology is both necessary and sufficient for most navigation tasks in built environments that feature distinct paths and places. It would be an interesting endeavour for future research to compare how knowledge of topological and metric relationships between places develops during cognitive mapping.

## Electronic supplementary material

Below is the link to the electronic supplementary material.

Supplementary file1 (DOCX 18 kb)
