# Supplementary material for: From repeating routes to planning novel routes: the impact of landmarks and ageing on route integration and cognitive mapping
Source: Psychol Res. 2020 Sep 14;85(6):2164–76. doi: 10.1007/s00426-020-01401-5 (PMC8357655; doi:10.1007/s00426-020-01401-5)
Supplement: Supplementary file 1 — Supplementary file1 (DOCX 18 kb) [file 426_2020_1401_MOESM1_ESM.docx]

Table Suppl.1. Comparison between identical and different Landmark Condition, for all participants (young and old)

| Age | | | | | MOCA | | | | | Gender | | |
| --- | --- | --- | --- | --- | --- | --- | --- | --- | --- | --- | --- | --- |
| Mean identical | Mean different | t | p | d | Mean identical | Mean different | t | p | d | #  identical | #  different | Fisher’s exact test p |
| 49.22 | 49.93 | t(117) =-0.16 | 0.87 | -0.03 | 26.78 | 26.71 | t(116.85)= 0.17 | 0.86 | 0.03 | 37f/24m | 32f/26m | 0.581 |

Table Suppl.2. Old participants: Comparison between finished vs. not finished experiment

| Age | | | | | MOCA | | | | | Gender | | |
| --- | --- | --- | --- | --- | --- | --- | --- | --- | --- | --- | --- | --- |
| Mean finished | Mean not finished | t | p | d | Mean finished | Mean not finished | t | p | d | #  finished | # not finished | Fisher’s exact test p |
| 71.30 | 69.85 | t(52.33) =1.39 | 0.17 | 0.32 | 26.26 | 25.35 | t(32.29)= 1.42 | 0.17 | 0.40 | 24f/23m | 15f/5m | 0.1042 |

Table Suppl.3. Old participants who finished experiment: Comparison between reached criterion (criterion+) vs. did not reach criterion (criterion-)

| Age | | | | | MOCA | | | | | Gender | | |
| --- | --- | --- | --- | --- | --- | --- | --- | --- | --- | --- | --- | --- |
| Mean criterion+ | Mean criterion- | t | p | d | Mean criterion+ | Mean criterion- | t | p | d | #  criterion+ | # criterion- | Fisher’s exact test p |
| 70.59 | 73.90 | t(11.31) = -1.54 | 0.15 | -0.68 | **26.81** | **24.2** | **t(14.23)= 3.79** | **0.0019*** | **1.35*** | 18f/19m | 6f/4m | 0.7238 |

Table Suppl.4. Comparison between identical and different for the different participant groups (old & young), subdivided by criterion (reached & not reached)

|  | Age | | | | | MOCA | | | | | Gender | | |
| --- | --- | --- | --- | --- | --- | --- | --- | --- | --- | --- | --- | --- | --- |
| Group | Mean identical | Mean different | t | p | d | Mean identical | Mean different | t | p | d | #  identical | #  different | Fisher’s exact test p |
| Old  criterion+ | 71.53 | 69.95 | t(26.01) = 1.05 | 0.30 | 0.37 | 26.20 | 27.23 | t(27.26) = -1.58 | 0.12 | -0.55 | 6f/9m | 12f/10m | 0.5077 |
| Old  criterion- | 73.00 | 75.25 | t(4.66) = -0.47 | 0.66 | -0.34 | **25.17** | **22.75** | **t(7.84) = 2.56** | **0.03*** | **1.54*** | 4f/2m | 2f/2m | 1 |
| Young  criterion+ | 22.15 | 21.96 | t(48.97) = 0.18 | 0.86 | 0.05 | 27.92 | 27.48 | t(48.83) = 0.87 | 0.39 | 0.24 | 16f/10m | 14f/11m | 0.7793 |
| Young  criterion- | 29 | N.A. | N.A. | N.A. | N.A. | 26 | N.A. | N.A. | N.A. | N.A. | 1f | 0 | N.A. |

Table Suppl.5. Performance (% correct; mean(SD)) by route type and participant group

|  | new route | | | retrace | | | repeat | | |
| --- | --- | --- | --- | --- | --- | --- | --- | --- | --- |
| LM condition | O- | O+ | Y+ | O- | O+ | Y+ | O- | O+ | Y+ |
| identical | 29 (14) | 53 (17) | 63 (18) | 43 (26) | 73 (19) | 83 (21) | 65 (16) | 74 (15) | 86 (19) |
| different | 31 (12) | 61 (14) | 81 (20) | 32 (32) | 76 (16) | 85 (20) | 55 (32) | 74 (16) | 87 (19) |

Table Suppl.6. Performance (blocks finished; mean(SD)) by participant group

| LM condition | O- | O+ | Y+ |
| --- | --- | --- | --- |
| identical | 5.50 (2.76) | 4.47 (1.84) | 3.27 (1.76) |
| different | 4.92 (3.22) | 3.68 (1.44) | 2.12 (1.43) |
